# COVID-19's Impact on the African American Community: A Stakeholder Engagement Approach to Increase Public Awareness Through Virtual Town Halls

**DOI:** 10.1089/heq.2020.0029

**Published:** 2020-07-15

**Authors:** Faith E. Fletcher, Shauntice Allen, Selwyn M. Vickers, Thomas Beavers, Christopher M. Hamlin, Dontrelle Young-Foster, Sherea Harris-Turner, Paul C. Erwin

**Affiliations:** ^1^ Department of Health Behavior and University of Alabama at Birmingham School of Public Health, Birmingham, Alabama, USA.; ^2^ Department of Environmental Health Sciences, University of Alabama at Birmingham School of Public Health, Birmingham, Alabama, USA.; ^3^ Office of the Dean, University of Alabama School of Medicine, Birmingham, Alabama, USA.; ^4^ New Rising Star, Birmingham, Alabama, USA.; ^5^ Tabernacle Baptist Church, Birmingham, Alabama, USA.; ^6^ 1917 Outpatient Clinic, University of Alabama at Birmingham School of Medicine, Birmingham, Alabama, USA.; ^7^ Housing Authority of the Birmingham District, Birmingham, Alabama, USA.; ^8^ Office of the Dean, University of Alabama School of Public Health, Birmingham, Alabama, USA.

**Keywords:** stakeholder engagement, COVID-19, virtual town hall, health inequities

## Abstract

COVID-19 has created a rapidly evolving public health crisis disproportionately impacting African Americans due to persistent inequities. The changing COVID-19 guidelines have resulted in concerns expressed by the American public, including unique concerns expressed by African Americans. To increase COVID-19-related awareness and dialogue among the African American community, the University of Alabama at Birmingham School of Public Health and the Housing Association of the Birmingham District convened a virtual town hall. This process of stakeholder engagement underscored the importance of cross-disciplinary expertise and collaboration and of community education and outreach by trusted sources.

## Introduction

COVID-19 has created a rapidly evolving public health crisis. This pandemic has required communities to promptly adhere to new recommendations put forth by the federal government and leading public health agencies to mitigate population health morbidity and mortality. The changing COVID-19 guidelines have resulted in concerns expressed by the American public, including unique concerns expressed by African Americans that are likely rooted in institutional distrust stemming from persistent legacies of inequities.^1,2^ Earlier reports, in fact, suggested skepticism surrounding African Americans' perceptions of susceptibility to COVID-19 infection. Several articles were published to shift misguided public perceptions, including a seminal article featuring Dr. Georges C. Benjamin, Executive Director of the American Public Health Association, and one of the nation's most influential physician leaders. Notably, Dr. Benjamin expressed: “We [African Americans] get a lot of misinformation circulating through our communities. We fundamentally don't trust some of the [non-black] institutions because they do not serve us well. We need to make sure our trusted institutions, clinicians of color, churches, community organizations, are better educated.”^3^ Indeed, increasing the delivery of health information and messages by trusted and respected stakeholders is critically important.

Numerous reports highlight that African Americans are at higher risk for both being infected with COVID-19 and, once infected, having worse outcomes.^3–7^ The high rates of service sector jobs (grocery clerks, bus drivers, building custodians), reliance on public transportation,^8^ and crowded housing conditions in some African American communities substantially contribute to the increased risk of acquiring COVID-19 among this population.^9,10^ Less access to care, delays in seeking care because of transportation and financial barriers, and greater levels of comorbidities (such as diabetes and hypertension) increase the likelihood of poor outcomes for African Americans who do get infected.^3,4,6^ African American leaders, social justice/grassroots organizations, and other influential stakeholders have led the national call for increased research, screening, testing, and race/ethnicity data to understand, document, and address COVID-19 inequities among African American communities.^4,5,9,11–13^

In light of the novelty of COVID-19, the most effective prevention strategies to date include hand washing, wearing face coverings, social distancing, and adhering to stay-at-home orders.^14^ However, racial/ethnic minority populations are more likely to live in densely populated areas due to residential housing segregation fundamentally rooted in institutional racism.^6,15,16^ Thus, practicing social distancing may be particularly challenging for certain segments of the U.S. population.^14^ In Birmingham, Alabama, the population of which is ∼70.5% African American, the Housing Authority of the Birmingham District (HABD)^17^ leadership and other local community leaders have expressed the following: (1) concerns for the health and well-being of public housing residents during the pandemic; and (2) the need for targeted education and messaging delivered by trusted leaders. To increase dialogue around COVID-19 with the African American community, the University of Alabama at Birmingham (UAB) School of Public Health partnered with HABD to convene a virtual town hall on April 4, 2020. This virtual town hall was the first in a series sponsored by UAB's School of Public Health to raise public awareness of the impact of COVID-19 on vulnerable communities.

## Approach

To reach the general public, including public housing residents, the virtual town hall was livestreamed via HABD's Facebook page and cross-posted on multiple UAB Facebook pages (i.e., University-wide page, School of Public Health and School of Medicine). We invited panelists/stakeholders who self-identified as African American and have strong community relationships and knowledge of the issues faced by residents living in housing communities. Notably, the pastors who participated in the town hall have church sites in proximity to the housing communities managed by HABD. To advertise the town hall, electronic flyers were circulated through various sources, including UAB websites (including those for the Schools of Public Health and Medicine, Institute for Human Rights, and Center for Clinical and Translational Science), social media (including platforms maintained by HABD, New Rising Star Church (NRS), and Tabernacle Baptist Church), local media, national list servs, and word of mouth. We used Zoom to broadcast the town hall via Facebook Live, and convened a practice meeting with moderators and panelists 24 h before the event to address any potential connectivity and/or technological issues. The 90-min town hall was recorded and made available via YouTube for individuals who were unable to attend and for ongoing education and dialogue.^18^ To date, this town hall has generated 48.6K views. The first half of the conversation focused on health, whereas the second half focused on the role of faith and community in the COVID-19 response. The town hall was moderated by two public health experts who self-identify as African American. Moderators and panelists utilized the Zoom Chat Box to communicate questions, suggestions, and challenges throughout the town hall.

## Panelists/Stakeholders^1^

**Dr. Selwyn M. Vickers** has been the Senior Vice President of Medicine and Dean of the UAB School of Medicine since October 2013 and is a world-renowned surgeon, pancreatic cancer researcher, and pioneer in health disparities research.

**Dr. Thomas Beavers** is the pastor of NRS.^19^ NRS is located in the Eastlake Community of Birmingham. Marks Village, a 500-unit apartment community operated by HABD, is less than a mile from NRS.

**Dr. Christopher M. Hamlin** is the pastor of Tabernacle Baptist Church.^20^ Tabernacle Baptist Church, located in the Smithfield Neighborhood, is less than half a mile from the 456-unit Smithfield Court Housing Community operated by HABD. Dr. Hamlin also serves as Chaplain/Education Specialist at UAB's 1917 Clinic, an HIV outpatient clinic.

**Mrs. Dontrelle Young-Foster** is the Interim President/CEO of HABD, which manages numerous public housing projects within Birmingham and provides housing assistance through administration of a Section 8 program.

**Mrs. Sherea Harris-Turner** is the Communications Director for HABD and is responsible for managing and directing HABD's external communications and promoting HABD's programs.

## Virtual Town Hall Content

The health segment focused broadly on COVID-19 prevention and symptomology, understanding chronic illness, and the impact of COVID-19 on the African American community. To address issues surrounding the susceptibility of African Americans to COVID-19, Dean Vickers stated: “Not only can we [African Americans] get it, but when we do get it, we tend to do much worse.” Dean Vickers also confirmed that COVID-19 infection stresses a person's body more than normal, and individuals with underlying health conditions, including chronic illness (e.g., heart disease or diabetes) may be especially affected.

The faith and community segment focused on alternative methods that churches are using to engage congregations, methods individuals can use to cope with death and dying, and resources available to the community. Dialogue regarding in-person church gatherings was motivated by reported outbreaks of COVID-19 linked to both church and funeral services in African American communities.^21^ Pastors Beavers and Hamlin suggested innovative techniques to promote social and spiritual connectedness while adhering to physical distancing guidelines and stay-at-home orders. In particular, technology has afforded churches throughout the world the opportunity to remain connected with their congregants during this pandemic. During the discussion, Pastor Beavers presented a compelling description of his personal experience with safely conducting a funeral for a loved one during the pandemic by leveraging technology. Pastor Beavers and his team prerecorded his grandfather's celebration of life service in segments. Singers, musicians, those who gave life reflections, and clergy arrived at different times to record their designated parts of the service, and the NRS video team combined the footage into one video. On the day of the homegoing, the service was livestreamed to the world. In addition, the NRS team placed LED video screens outside the church so people could drive in and watch the service. Through the gift of technology, Pastor Beavers and his family were able to honor his grandfather's legacy. In addition, Mrs. Dontrelle Young-Foster and Mrs. Sherea Harris-Turner stressed the importance of residents utilizing multiple methods of communication (e.g., text alerts, websites, social media, office phone lines, and drop boxes) to access up-to-date information about COVID-19 and other essential resources. See Table 1 for a summary of virtual town hall questions and responses.

**Table 1.** Summary of virtual town hall questions and responses

Questions	Responses
Healthcare	
• How is this different from the flu, and why should the public be proactive?	• They are similar in that both have a high infection rate and are most frequently transmitted through respiratory droplets. They are also similar in that most people will recover.
	• They are different in that there are no proven drugs to treat COVID-19, but there are drugs for the flu. Studies are underway for drugs to treat COVID. Also, the mortality rate for COVID-19 is higher than the flu because COVID infections go deeper in the lungs and are more likely to cause damage.
	• However, this is not a direct parallel to the early days of HIV epidemic. Although there is no treatment for COVID-19, most people will survive without treatment.
• How can we protect ourselves and others from getting this virus?	• There are steps for both health care and community.
	• Health care—work to find treatments and expand testing to more individuals, particularly asymptomatic ones.
	• Community—Limit the spread of COVID-19 by maintaining 6 foot distance from others, wearing masks or face coverings outside the home, avoiding gatherings outside the home, and washing your hands after touching anything from outside your home.
• What do the symptoms of COVID-19 feel like? When should someone be concerned and seek medical advice?	• Common symptoms of COVID-19 infection include high fever, fatigue, a constant dry cough, and shortness of breath.
	• When shortness of breath gets severe you can't walk a short distance without distress, you should seek care. If you have an underlying chronic disease, you might not want to wait until shortness of breath is severe.
• What does it mean to have a chronic disease?	• A chronic disease is any condition that doesn't go away or can't be cured (e.g., heart disease or diabetes). However, many chronic diseases can be managed to the point that they don't interfere with your daily life.
	• COVID-19 infection stresses a person's body more than normal, and individuals with a chronic disease may have a harder time fighting that.
• What does COVID-19 mean for children and African Americans?	• Although children are less likely to have severe symptoms, they can be carriers and spread COVID-19.
	• New evidence from other big cities shows that African Americans are being infected with and dying from COVID-19 at disproportional rates compared to other races.
	• This may be due to higher rates of chronic disease and lack of access to health care.
Faith and community organizations	
• What alternative methods are churches using to engage congregations?	• It is important to remember that we must physically distance from each other, but we can still connect socially and spiritually in new ways.
	• Congregations are doing virtual worship services through Zoom and conference calls, holding prayer lines on Facebook Live, and using congregation mass texting systems to provide up to date information.
	• A church isn't the building, people are the church. Although buildings are closed, the church isn't.
• How is the Housing Authority helping its residents?	• They are trying to utilize multiple methods of communication (test alerts, website, social media, office phone lines, drop boxes) to ensure every resident can access up to date information about resources.
	• Housing authority wants people to reach out and let them know if their needs or circumstances are changing so they can provide assistance.
	• Contact your housing office if you think you need to get tested and can't access it.
	• Transportation to testing sites is available through partner organizations.
	• Local agencies are looking into mobile testing sites to go directly to communities in need.
• How do families cope with changes in funeral services?	• Changes pertain to death and dying from COVID *and* other causes.
	○ Memorial services can't happen
	○ Inability to visit family members in the hospital
	• Some individuals more open to cremation than before.
	• Although in person memorial services can't happen, some are exploring pre-recorded funeral services, drive up services or streaming services.
• What can the community do to help?	• Be good neighbors by staying positive and connected, even while distanced,
	• Follow local and state health guidelines and stay at home if possible.
	• Participate in https://www.helpbeatcovid19.org/ even if you're healthy.
Audience questions	
• How can someone distinguish between asthma symptoms and COVID-19?	• With COVID-19 you'll have additional symptoms of fever and full-body fatigue.
	• Asthma is a temporary reaction in your airway, while COVID-19 causes more immediate damage.
• What should someone do if they are stopped by police while out for an essential trip?	• Officers are not looking to arrest anyone, but might remind people about social distancing for safety.
• Do some people have a genetic predisposition for COVID-19?	• There is no evidence of that right now, but we still don't understand why some people are infected and asymptomatic, while others aren't.
• Is there a vaccine available?	• Development is underway, but it takes a lot of work to develop one that is both safe and effective.
	• Usually takes 12–18 months to get a vaccine ready for patients.
• Does UAB have enough ventilators? How will hospitals decide who to treat if they are overcrowded?	• Right now UAB is nowhere near full capacity (because they have stopped nonessential medical services) and has enough ventilators, as well as others they can bring out of storages, and plans to possibly acquire more.
	• There are good algorithms to understand how badly someone needs care. However, it's harder to know which patients are more likely to benefit from care.
	○ Decisions about rationing care are made by teams to prevent bias and disparities in who does get care (i.e., racial disparities, people w/o insurance)

UAB, University of Alabama at Birmingham.

## Lessons Learned

### Cross-disciplinary expertise and collaboration

COVID-19 is a public health crisis that requires knowledge and expertise from multiple disciplines (e.g., public health, medicine, community, and faith-based institutions) to provide comprehensive, accurate, and relevant information to the public. Innovative ways of thinking and engaging individuals are also essential to safely navigate this pandemic. For example, the importance of using technology to support faith communities was reiterated by an audience member who stated that faith leaders and technology experts—including those from historically black colleges and institutions and minority technology companies—must partner to deploy technology solutions (Table 2).

**Table 2.** Comments from live Facebook feed

Webinar topic	Selected viewer comments
• Comorbid conditions and COVID-19	• The numbers were well over 60% last week. They've referred to it as a crisis in a crisis. We are tracking all black deaths on Truth Tellers Covid19. We are asking for stories and statistics. It's astonishing. Michigan & Virginia also publish race data.
	• Thank you for saying heart disease from @blackheartassociation
	• Very well explained!! Chronic illnesses!!
• Racial disparities in COVID-19 infection and mortality rates	• We need to watch more of this for other marginalized populations and intersecting identities… younger black men, for example, have been all over my timeline.
	• The disproportionate impacts on AA is very disturbing.
	• There also needs to be testing for African Americans specifically. As we have learned over the years, ACE inhibitors and statins don't agree with most of the African American populations and alternatives should be found. Same with the COVID-19 remedy.
• Religion during COVID-19	• As the church, ministry is and should be paramount during this time in the effort for meeting the needs of the least of these. Even in social distancing we have tried to meet those needs. Their plight did not go away because of this covid-19 virus, but has become rather magnified in it.
	• COVID-19 and all health equity issues from a biblical context is “Least of these…” when we center those at most risk… we solve for those who are privileged.
• Using technology to connect faith communities	• VERY good point—finding ways to leverage technology to reach congregations. we need that for sure.
	• Amen! I agree with physically distancing and not socially.
	• Technology is definitely a blessing!! However, can be a curse as well!!!
	• Church leaders must reach out to technology experts in their communities to deploy technology solutions. Experts from HBCUs, minority technology startups and consultants in our communities have the ability to assist and coach. We are clear: This is the new norm and technology will be a central tool moving forward.
• Additional questions/comments	• What are we doing to help the homeless population?
	• What is the difference in the breathing and cough if you have asthma?
	• Police brutality is truly the biggest disgrace in American history. Through this pandemic we are already making changes. Remember we must follow the right leaders.

### Community education and outreach

Ethical engagement of communities during a pandemic calls for institutional support to meet the needs of local communities, especially communities burdened by persistent health and economic inequities. Justice, which is central to the mission of public health, calls for improving the availability, accessibility, and acceptability of COVID-19 information and testing, particularly among the most disadvantaged.^22^ As a follow-up to the town hall, the School of Public Health created short educational clips from the town hall to assist HABD with ongoing resident education. UAB's Minority Health and Health Disparities Research Center also provided HABD with COVID-19 prevention and testing information.^23^

### Reliable information from trusted sources

In light of the influx of information and the inherent difficulty in identifying reliable sources of information, it is important to engage trusted sources to disseminate accurate information to communities. African American pastors, health care providers, public health experts, and other community leaders are well-poised to deliver COVID-19 information and messages through various communication channels, including via livestreaming social media platforms that facilitate a two-way dialogue between the speaker and the audience. To address misconceptions surrounding COVID-19, the town hall panelists emphasized that African Americans are, in fact, infected with and dying from COVID-19 at disproportionate rates, and adhering to public health recommendations is currently the best line of defense.

### Considerations for future virtual town halls

Next steps include establishing an evaluation framework to systematically assess the user experience and elicit feedback regarding future town hall content and format. Preliminary feedback and comments from the live Facebook feed (Table 2) suggest overall satisfaction with the town hall. Recommendations for future town halls included highlighting other marginalized populations with intersecting identities who are adversely impacted by COVID-19-related inequities. Thus, a second town hall focused on the impact of COVID-19 on women, and a third town hall focused on housing and food insecurity imposed by the pandemic. While Facebook appears to be an appropriate platform, especially given HABD's and our faith-based partners' established Facebook pages and ongoing social media communication, it is important to explore other methods to reach persons who may not have access to computers and/or the Internet. Despite limitations associated with inadequate access to technology among marginalized populations, information delivered through social media platforms can reach nonsocial media users through other communication channels (e.g., word of mouth).

## Conclusion

The COVID-19 pandemic underscores the importance of long-standing academic/community partnerships to appropriately meet the needs of diverse communities in a crisis.^24^ Notably, our initiative relied on existing relationships to conceptualize, plan, and execute the virtual town hall in 7 days. In the time of COVID-19, some Americans are faced with navigating concomitant life demands caused by systemic racism. As conveyed by Dr. Martin Luther King Jr, the “inescapable network of mutuality” reminds us of our interconnectedness as humans and our duty to work collectively to seek justice in health by centering the most marginalized for the sake of the common good.

## Abbreviations Used

HABD: Housing Authority of the Birmingham District
NRS: New Rising Star Church
UAB: University of Alabama at Birmingham

## Notes

^1^ Consistent with a stakeholder-engagement approach, panelists are also authors of this article.
